# The Effect of Silane Acrylate Containing Ethylene Glycol Chains on the Adhesive Performance and Viscoelastic Behavior of Acrylic Pressure-Sensitive Adhesives for Flexible Displays

**DOI:** 10.3390/polym15173601

**Published:** 2023-08-30

**Authors:** Woong Cheol Seok, Jong Tae Leem, Ho Jun Song

**Affiliations:** Green and Sustainable Materials R&D Department, Korea Institute of Industrial Technology, 89 Yangdaegiro-gil, Ipjang-myeon, Seobuk-gu, Cheonan-si 31056, Chungcheongnam-do, Republic of Korea; vmfosel07@naver.com (W.C.S.); netwt700@naver.com (J.T.L.)

**Keywords:** acrylic pressure-sensitive adhesive, optical clear adhesive, adhesion properties, viscoelastic behavior

## Abstract

In this study, novel silane acrylates, such as diethylene glycol diacrylate (DEGDA) and tetraethylene glycol diacrylate (TEGDA), containing ethylene glycol chains were synthesized and introduced into acrylic pressure-sensitive adhesives (PSAs) to regulate their peel strength and rheological properties. The synthesized silane acrylates effectively improved the cohesion and adhesive properties of the acrylic PSAs, even with only 1 wt% addition. In addition, the glass transition temperature and flexibility of acrylic PSAs were also affected by the increase in free volume induced by ethylene glycol chains. The silane acrylates also improved the viscoelasticity of the acrylic PSAs, which exhibited excellent recovery (62–96%) and stress relaxation (>90%) properties owing to the increased elasticity. Additionally, the acrylic PSAs prepared with the silane acrylates showed excellent optical properties (transmittance ≥ 90%, haze ≤ 1%) and exhibited behavior suitable for application in flexible displays from a comprehensive perspective.

## 1. Introduction

Photocurable acrylic pressure-sensitive adhesives (PSAs) are polymerized in bulk form without a solvent, and the polymerization and curing processes are performed under ultraviolet (UV) irradiation. Thus, the reaction rate is rapid and the process is simple, which is highly advantageous in terms of equipment, productivity, and energy efficiency compared to conventional thermal polymerization. In addition, because low-temperature curing is possible and there is no solvent or unreacted monomers after the reaction, the process has a wider industrial application scope than solvent-type polymerization [1,2,3]. In the display industry, photocurable acrylic PSAs are extensively used as optically clear adhesives (OCAs) and function by attaching each layer of the display, such as the cover window, touch panel, polarizers, and light-emitting layer. Owing to the recently increasing interest in different flexible display devices such as curved, foldable, and rollable displays, OCAs require various characteristics, including display layer fixation, optics, adhesiveness, and viscoelastic behavior [4,5].

When strain is applied to a flexible display, stress and strain influence each display component because, generally, OCAs exhibit low flexibility, causing various defects upon deformation. Therefore, applying OCAs to flexible displays requires viscoelastic properties such as flexibility, recovery and relaxation characteristics, and adhesive strength [6]. Therefore, several studies have been conducted to improve the recovery and stress relaxation properties of OCAs by controlling their crosslinking density [7], patterning [8], and blending with highly elastic elastomers [9,10]. However, because these physical properties are complementary, it is difficult to satisfy all the related properties. Thus, continuous research is required to bridge the gap in the literature on the correlation between viscoelastic behavior and the recovery and stress relaxation properties of OCAs.

We have designed and synthesized new acrylates with different shapes and functions, and various studies have been conducted to improve the morphology and viscoelastic properties of these acrylic PSAs. Previously, we have reported the effect of ethylene glycol chains on the morphology and rheology of acrylic PSAs [11]. Ethylene glycol chains affected the free volume between the main chains, which resulted in excellent flexibility, even at a cryogenic temperature of −40 °C, and excellent viscoelasticity and adhesive properties by realizing high cohesion characteristics via physical crosslinking in the absence of a crosslinker. In addition, a new acrylate was synthesized by introducing a silane group into urethane acrylate, and the adhesive strength and viscoelasticity of the acrylic PSA was regulated using the bulky characteristics of the urethane bond and silane group [6]. Silicon compounds have better mechanical and thermal properties than organic materials composed of carbon. In addition, the Si–O–Si bonding angle is 140 to 180, which is higher than the C–O–C ether bond angle of 110; thus, it has high flexibility. Due to these excellent properties, it is applied as a polymer material in various fields, from industrial films and point adhesives to surface coatings/hard coatings. Recently, silane/siloxane compounds have been studied for their application as functional hard coating materials applicable to flexible displays due to their high hardness and flexibility [12,13]. Consequently, the novel silane acrylate improved the cohesion and elasticity of the corresponding acrylic PSA, even with only 1 wt% addition, and showed high recovery characteristics and stress relaxation behavior, even at 400% deformation. In addition, it exhibited excellent flexibility because it did not significantly affect flexibility owing to its low content, and excellent adhesive properties were achieved owing to the improved cohesion. 

In this study, new silane acrylates, diethylene glycol diacrylate (DEGDA) and tetraethylene glycol diacrylate (TEGDA), are synthesized using ethylene glycol chains. In addition, the various effects of the new silane acrylates on the physical properties of the corresponding acrylic PSAs are investigated. The chain length of the new silane acrylate is controlled according to the ethylene glycol group, and a silane group is introduced at the end to compensate for the low cohesion of the acrylic PSA and the modulus at high temperatures. The obtained silane acrylate is mixed with 2-ethylhexyl acrylate (EHA) and 2-hydroxyethyl acrylate (HEA) to synthesize the flexible acrylic PSAs by UV irradiation. The adhesive and viscoelastic properties of the synthesized acrylic PSAs are analyzed, and the effect of the silane acrylate chain length on the flexibility of the acrylic PSAs is evaluated. In addition, the correlation between the silane acrylate and the adhesive strength is investigated, and the applicability of the acrylic PSAs in the flexible display field is evaluated based on their optical properties and recovery and relaxation characteristics.

## 2. Materials and Methods

### 2.1. Materials

EHA (99%), HEA (95%), DEGDA, and TEGDA were purchased from TCI (Tokyo, Japan). (3-mercaptopropyl)trimethoxysilane (MPTMS; 95%) was purchased from Sigma–Aldrich (St. Louis, MO, USA), and hexylamine (HA; 99%) was purchased from Acros Organics. Irgacure 184, which is a photoinitiator, was purchased and used by Shinyoung Radchem. 1-dodecanethiol (98%), which is a chain-transfer agent, was purchased from Alfa Aesar. All materials were used without purification.

### 2.2. Synthesis of Silane Acrylates Containing Ethylene Glycol Chains

#### 2.2.1. Diethylene Glycol Silane Acrylate (DEGSA)

DEGSA was synthesized via the thiol-ene reaction of di-functional acrylate and thiol-silane. DEGDA (10 g, 46.7 mmol) was placed in a 250 mL one-neck round bottom flask and MPTMS (9.17 g, 46.7 mmol) was slowly added dropwise. Subsequently, HA (0.55 wt% of the total weight) was added as a catalyst, and the reaction mixture was stirred at room temperature for 4 h. After stirring, the crude product was purified through column chromatography (SiO_2_, HEX:EA = 1:1) to remove the catalyst, and the final product was obtained in a transparent and slightly viscous liquid form (18.69 g, yield: 97.55%). Proton nuclear magnetic resonance (^1^H NMR; 300 MHz, CdCl_3_, Me_4_Si): ^1^H NMR (300 MHz, CdCl_3_, Me_4_Si): δ = 6.44–6.38 (d, 1H), 6.18–6.09 (d, 1H), 5.84–5.81 (d, 1H), 4.32–4.22 (m, 4H), 3.75–3.68 (m, 4H), 3.54 (s, 9H), 2.76 (t, 2H), 2.61 (t, 2H), 2.53 (t, 2H), 1.68 (q, 2H), 0.72 (t, 2H), Anal. Calc. for C_16_H_30_O_8_SSi: C, 46.81; H, 7.37; O, 31.18; S, 7.81. Found: C, 46.73; H, 7.42; O, 23.6; S, 7.94.

#### 2.2.2. Tetraethylene Glycol Silane Acrylate (TEGSA)

TEGSA was synthesized using the same process as used for DEGSA. The synthesized TEGSA was also purified through column chromatography (SiO_2_, HEX:EA = 1:1) and was obtained as a product in the form of a transparent, slightly viscous liquid (16.25 g, yield: 98.53%). ^1^H NMR (300 MHz, CdCl_3_, Me_4_Si): δ = 6.43–4.39 (d, 1H), 6.19–6.10 (d, 1H), 5.85–5.81 (d, 1H), 4.31 (t, 2H), 4.24 (t, 2H), 3.75–3.66 (m, 12H), 3.54 (s, 9H), 2.74 (t, 2H), 2.62 (t, 2H) 2.54 (t, 2H), 1.74–1.67 (q, 2H), 0.74 (t, 2H), Anal. Calc. for C_20_H_38_O_10_SSi: C, 48.17; H, 7.68; O, 32.08; S, 6.43. Found: C, 47.75; H, 7.58; O, 25.61; S, 6.68.

### 2.3. Fabrication of Acrylic PSAs

Acrylic PSAs were fabricated according to the following procedure: EHA, HEA, and the synthesized silane acrylates (DEGSA and TEGSA) were added to a three-neck round bottom flask using the same ratio as listed in Table 1, and then, irgacure 184 (0.5 wt%) and 1-dodecanethiol (0.025 wt%) were added. After 30 min, UV irradiation was conducted using a UV lamp (black light, 10 W) installed on the outside of the flask and under a nitrogen atmosphere to polymerize the acrylic pre-polymers. The synthesized acrylic pre-polymers were uniformly applied between the release and polyethylene terephthalate (PET) films at a thickness of 50 µm. Finally, the acrylic PSAs were fabricated by UV irradiation (2000 mJ/cm^2^).

### 2.4. Characterization

The structures of synthesized silane acrylates were analyzed using the 1H NMR spectrum obtained by Fourier-transform nuclear magnetic resonance (FT-NMR; Bruker, Billerica, MA, USA, AVANCE III, 300 MHz), and CdCl_3_ was used as the solvent. The element compositions of the novel silane acrylates were analyzed by elemental analysis (EA; Thermo Fisher, Waltham, MA, USA, Flash 2000). The molecular weights of the acrylic pre-polymers were measured using gel permeation chromatography (GPC; Futecs (Daejon, Republic of Korea), P-4000) with an infrared (IR) detector. The column temperature was set at 40 °C, and high-performance liquid chromatography (HPLC)-grade tetrahydrofuran was used as the eluent. Polystyrene with a single molecular weight was used as the molecular weight standard.

The crosslinking degrees of the acrylic PSAs were measured based on the gel fraction, which was determined by the solvent solubility. The acrylic PSAs were immersed in toluene and maintained at room temperature for 1 d. Thereafter, the dissolved portion was removed by filtration, and the residual solid was dried at 80 °C. The gel fractions of the acrylic PSAs were calculated using the following equation:

Gel fraction (%) = (W_1_/W_0_) × 100, (1)

where W_0_ and W_1_ represent the weight of the acrylic PSAs before and after filtering, respectively.

The glass transition temperature (T_g_) values of the acrylic PSAs were measured by differential scanning calorimetry (DSC; PerkinElmer, Waltham, MA, USA, DSC 8000), and each sample was measured in the temperature range of −50–200 °C at a heating rate of 10 °C/min under a nitrogen atmosphere.

The surface wettability of the acrylic PSAs was measured using a contact angle analyzer (SEO, Phoneix (Daejon, Republic of Korea)), and the hydrophilicity of the surface by silane acrylates was measured using a contact angle analyzer, and the hydrophilicity of the acrylic PSA by silane acrylate was evaluated through the angle of the droplet formed on the surface.

The peel strength of the acrylic PSAs were measured using a peel tester (Chemilab, SurTa). Each measurement sample was prepared by attaching the acrylic PSA to a polyimide film (25 × 200 mm) and a rubber roller with a load of 2 kg repeatedly two to three times. The prepared sample was maintained at room temperature for 30 min, and the peel strength was measured at a tensile rate of 300 mm/min in the 180° peel mode. The peel strength were measured repeatedly five to seven times, and the average value was recorded.

The viscoelastic behavior of the acrylic PSAs was analyzed using a rheometer (Anton Paar, Tokyo, Japan, MCR 102). The acrylic PSAs were coated to a thickness of approximately 500 µm using a mold, followed by UV irradiation at 2000 mJ/cm^2^ to produce the samples in a free-standing form without support. The fabricated samples were fixed on the rheometer plate and subjected to 0.4% strain and an oscillation frequency of 1 Hz, and then measured in the temperature range of −40–100 °C at a constant heating rate of 4 °C/min.

The creep and stress relaxation tests were also performed using a rheometer (Anton Paar, MCR 102). The measurement samples were prepared in the same shape as that of the viscoelasticity measurement samples. Each sample was fixed to the rheometer plate. The creep characteristic was measured to determine the strain change over time by applying a stress of 10,000 Pa to the sample for 10 min.

Stress relaxation was measured to determine the stress change over time by applying 100% and 400% strain to the sample for 10 min, respectively. Subsequently, the applied strain was removed and recovered for 10 min. The recovery characteristics and stress relaxation were evaluated based on the deformation of the stress and strain of the sample, and the results were calculated using the following equation:

Recovery characteristic (%) = (S_0_ − S_t_/S_0_) × 100, (2)

where S_0_ is the strain at the onset of recovery (t = 0 s) and S_t_ is the recovered strain at t = 300 s;

Stress relaxation (%) = (F_i_ − F_t_/F_i_) × 100, (3)

where F_i_ and F_t_ represent the initial and final stresses, respectively, under a constant strain.

The final optical properties of the acrylic PSAs were evaluated by transmittance and haze. The transmittance was analyzed by ultraviolet–visible (UV–Vis) spectrometry (GBC, Cintra 10e). The background was recorded using a bare PET film, and transmittance was measured in the wavelength range of 400–800 nm. The haze was analyzed using a haze meter (SUGA Test Instruments, HZ-V3 (Tokyo, Japan)).

## 3. Result and Discussion

### 3.1. Characterization of Silane Acrylates containing Ethylene Glycol Chains

**Scheme 1:**
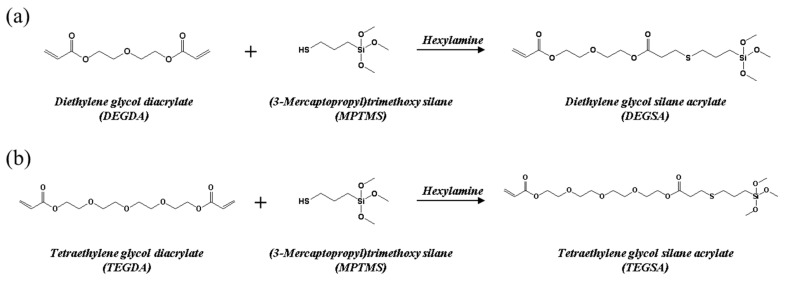
Novel silane acrylate based on ethylene glycol—(**a**) DEGSA, (**b**) TEGSA.

Novel silane acrylates (DEGSA and TEGSA) were synthesized via an amine-catalyzed thiol-ene reaction, and the synthesis process is illustrated in Scheme 1. The thiol-ene reaction is also called the thiol Michael reaction, and the combination of thiol and unsaturated compounds is carried out under the catalysis of nucleophiles or primary amines. Hexylamine used to synthesize DEGSA and TEGSA was also applied as a primary amine catalyst. The structure of the synthesized silane acrylates was analyzed by FT-NMR, and the results are shown in Figure S1. Ethylene glycol-based diacrylates DEGDA and TEGDA reacted with the thiol group of MPTMS to break the symmetrical structure, and the electron density of ethylene glycol and the aliphatic chain of silane was rearranged by sulfur, causing the proton peak to shift upfield. In addition, it was confirmed that the new silane acrylates DEGSA and TEGSA were successfully synthesized by calculating the proton integral ratio.

Acrylic pre-polymers were synthesized by mixing EHA, HEA, and the novel silane acrylates DEGSA and TEGSA, as listed in Table 1. The molecular weights of the synthesized acrylic pre-polymers were measured by GPC, and the results are listed in Table 2. The acrylic pre-polymer without the novel silane acrylates has a molecular weight of 870,000 g/mol. In contrast, upon silane acrylate addition, the molecular weight increased within the range of 1,110,000–1,440,000 g/mol. This is because the synthesized silane acrylate has a relatively high free volume due to the presence of the ethylene glycol chains (-(CH_2_CH_2_O)-), silane aliphatic chains (-CH_2_-), and bulky terminal silane groups [14,15]. The introduction of long-chain silane acrylates increased the distance between the polymer backbones of the acrylic PSA and the increased free volume shortened the residence time of the polymer during the GPC. Thus, the acrylic pre-polymer prepared with the long-chain silane acrylates exhibited a higher molecular weight than that prepared without the long-chain silane acrylate. Additionally, the synthesized silane acrylates have a relatively high molecular weight (DEGSA: 410.56 g/mol, TEGSA: 484.63 g/mol), which is presumed to be responsible for the increase in the molecular weight of the acrylic pre-polymer. 

**Figure 1:**
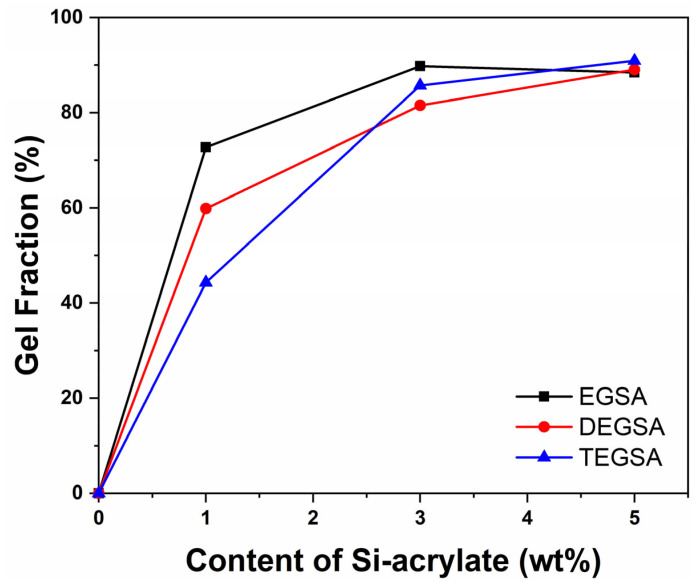
Gel fraction of synthesized PSAs with novel silane functional derivative.

The gel fraction was measured to evaluate the crosslinking behavior of the acrylic PSAs, and the results are shown in Figure 1. The acrylic PSA without silane acrylate was completely dissolved in the solvent, and the gel fraction was not measured. All the acrylic PSAs did not use a multifunctional crosslinking agent and, thus, a crosslinking network was not formed [16,17]. It was, therefore, inferred that the backbone of the acrylic PSA without silane acrylate was simply entangled. When 1 wt% of silane acrylate was added to the acrylic PSAs, the gel fraction increased to more than 40%. These results were owing to the ethylene glycol chains in the novel silane acrylates. We previously demonstrated that the ethylene glycol side chain plays a role in increasing chain entanglement to obtain flexibility and can undergo physical crosslinking due to interchain secondary bonding by the oxygen atoms of ethylene glycol [11]. Therefore, the gel fraction increased rapidly because the long chains of the novel silane acrylates result in the physical crosslinking of the acrylic PSAs. In addition, because the bulky silane end group also contributes to the increase in the cohesive force of the polymer chain, it was assumed that it also improves the gel fraction. Comparing DEGSA and TEGSA, the chain length of silane acrylate also affected the gel fraction of the acrylic PSA (DEGSA-acrylic PSA: approximately 60%, TEGSA-acrylic PSA: approximately 40%). These results may be explained by the packing density of the polymer chain [14]. DEGSA has a shorter ethylene glycol chain length than TEGSA, and its packaging is closer owing to chain entanglement. Thus, it is difficult for the solvent to penetrate into the polymer chain and cause swelling. In contrast, the longer ethylene glycol chain length in TEGSA results in a looser packing density. Because loose packing allows for easy solvent penetration, unreacted and low-molecular-weight polymers in the swollen chain can be easily dissolved. Therefore, the chain length of the silane acrylate controlled the free volume of the acrylic PSA. The shorter chain length of DEGSA suppressed solvent penetration, resulting in a higher gel fraction than that of TEGSA. When more than 3 wt% of the new silane acrylate was added, the acrylic PSAs exhibited a similar gel fraction and were not significantly affected by the chain length. These results may be explained by the degree of chain entanglement and crosslinking density. When the silane acrylate content was as low as 1 wt%, the ratio of side chain entanglement was low, and the free volume of the polymer chain depended on the ethylene glycol group owing to the low physical crosslinking density. In contrast, at a silane acrylate content of 3 wt% or higher, a high crosslinking density was observed due to increased chain entanglement, and a similar free volume was obtained independent of the acrylate chain length. Therefore, when the acrylate content was more than 3 wt%, the acrylic PSAs formed via physical crosslinking exhibited similar free volumes and, thus, a similar gel fraction was obtained independent of the silane acrylate chain length.

### 3.2. Thermal Properties of the Acrylic PSAs

**Figure 2:**
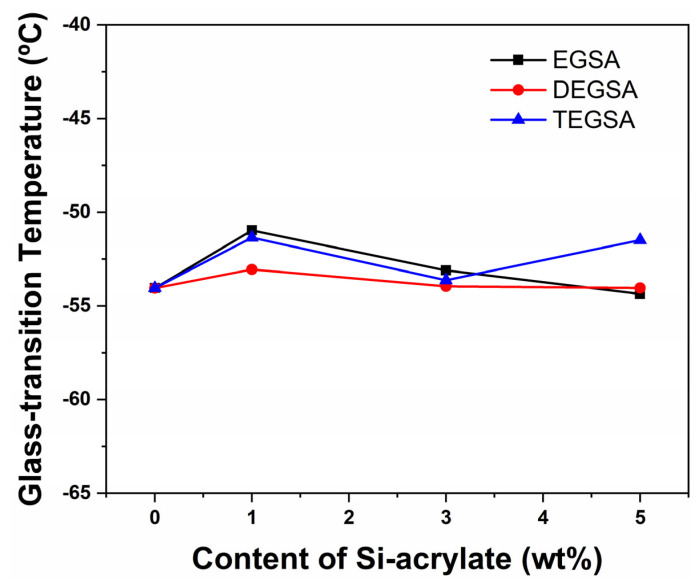
The glass transition temperature of synthesized PSAs.

The effect of the synthesized silane acrylate on the T_g_ of the acrylic PSA was evaluated by DSC, and the results are presented in Table 2 and Figure 2. The acrylic PSA without silane acrylate had a very low T_g_ (−54.05 °C). When 1 wt% silane acrylate was added, the T_g_ of the acrylic PSA was higher than that without silane acrylate because the chain mobility is limited by the chain entanglement and bulk silane groups in silane acrylate [18,19]. In addition, the increase in the T_g_ for the acrylic PSAs differed depending on the silane acylate structure, which is thought to be owing to the flexibility induced by the chain length and the characteristics caused by secondary bonding. When the silane acrylate content was increased to 3 wt%, the T_g_ decreased significantly. This is because the acrylic PSA backbone is farther apart when using the long-chain silane acrylate, resulting in an increase in free volume. The T_g_ of a polymer is greatly influenced by mobility, which is dependent on the free volume [15]. Silane acrylate has a long chain length owing to the ethylene glycol group and bulky characteristics owing to the silane group. Therefore, the introduction of silane acrylate increases the free volume of the acrylic PSA, which increases the mobility of acrylic PSA, and thus the T_g_ decreases. This result was observed, even when the silane acrylate content was 5 wt%, and similarly, the free volume of acrylic PSA was increased by silane acrylate addition. In particular, TEGSA increased the T_g_ of the acrylic PSA using a silane acrylate content of 5 wt% or higher, which is thought to be owing to the chain length of TEGSA. As a polymer chain lengthens, the T_g_ decreases owing to the soft characteristics of the polymer, but beyond a certain chain length, the flexibility decreases owing to entanglement or self-arrangement. TEGSA has the longest chain length among the silane acrylates, and because flexibility decreases owing to the above-mentioned factors, it is assumed that the T_g_ of the acrylic PSA increased when the silane acrylate content was 5 wt% or higher.

### 3.3. Adhesion Performance of the Acrylic PSAs

**Figure 3:**
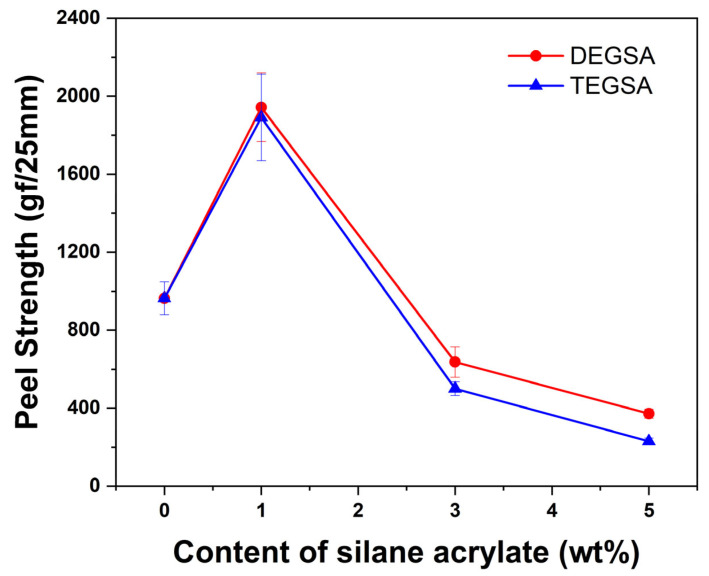
Peel strength of synthesized PSAs.

The adhesive properties of the acrylic PSAs prepared with silane acrylate were evaluated using a 180° peel test, and the results are shown in Figure 3. The acrylic PSA without silane acrylate has a peel strength of 960 gf/25 mm, but cohesive failure occurred owing to the absence of hard segments and crosslinking. When 1 wt% silane acrylate was introduced into the acrylic PSAs, the peel strength of the acrylic PSAs increased by approximately 100%; this is because the cohesion of acrylic PSA is improved by silane acrylate. 

As shown in Figure 1, long-chain silane acrylates induce an increase in chain entanglement and play a role in improving the cohesive properties of acrylic PSAs owing to bulky silane groups. This causes the peel strength of the acrylic PSA to increase rapidly, and clean peeling without residue was observed in the form of adhesive failure due to the improved cohesive force. Interestingly, the DEGSA- and TEGSA-acrylic PSAs exhibited different gel fractions but similar peel strengths. This is presumed to be owing to the surface hydrophobicity caused by the lengths of the side chains and ethylene glycol groups. As shown in Figure S2, TEGSA increased the surface hydrophilicity of acrylic PSA compared to DEGSA. This is because TEGSA has abundant oxygen atoms in its long ethylene glycol chains. The improved hydrophilicity makes the acrylic PSA more easily wetted on the substrate surface, which enhances the peel strength [20]. Owing to the complex action of the gel fractions and surface properties, the synthesized acrylic PSAs prepared with 1 wt% silane acrylate have similar peel strengths independent of the type of silane acrylate. At 3 wt% silane acrylate, the peel strength of the acrylic PSA decreased significantly. The same trend was observed at 5 wt% silane acrylate content. This is because the cohesive properties of the acrylic PSA increased rapidly as the silane acrylate fraction in the acrylic PSA increased. As observed in Figure 1, when more than 3 wt% silane acrylate is introduced into the acrylic PSA, a high gel fraction of approximately 80% was achieved independent of the silane acrylate type and, thus, the cohesion property also increased rapidly. A rapid increase in cohesive force causes a decrease in peel strength because the interaction between the acrylic PSA chains is stronger than that between the acrylic PSA and the substrate [21].

### 3.4. Viscoelastic Properties

**Figure 4:**
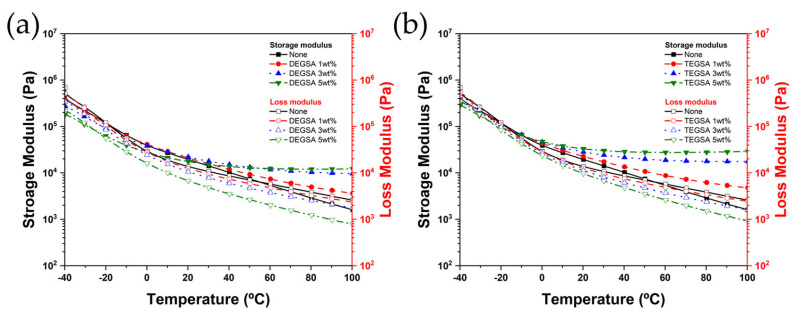
Storage modulus—(**a**) DEGSA, (**b**) TEGSA.

The viscoelastic properties of the silane acrylate-based acrylic PSAs were measured using a rheometer, and the viscoelastic behavior was evaluated based on the storage modulus (G′) and loss modulus (G″). As shown in Figure 4, all acrylic PSAs exhibited a low modulus of less than 10^6^ Pa in the low-temperature region, and the modulus gradually decreased with increasing temperature. Among them, the modulus of the acrylic PSA without silane acrylate decreased rapidly with increasing temperature, which is owing to the considerably low deviation between G′ and G″. G′ and G″ represent the elasticity and viscosity of a polymer, respectively, and G′ should be superior to G″ for a polymer to be used as a PSA [22]. When the deviation between G′ and G″ is low, a polymer exhibits liquid-like behavior because it is very soft and has a low cohesive force. Thus, as temperature increases, a point is reached where G′ and G″ intersect (G′ = G″, 54.72 °C), and beyond this point, it is difficult to use as a PSA because it behaves as a viscous liquid. The introduction of silane acrylates improved the modulus of the acrylic PSAs and showed a stable G′, even with increasing temperature. This is also thought to be owing to the improved cohesion by physical crosslinking with the silane groups of the silane acrylates. The ethylene glycol chains of silane acrylate undergo physical crosslinking by polymer entanglement, and the silane groups are bulky and have excellent heat resistance. Because these factors improve the elasticity, heat resistance, and mechanical properties of the acrylic PSA, stable viscoelastic behavior was obtained, even at high temperatures. These results were also confirmed by the deviation of G′ and G″ for the acrylic PSAs (G′ >> G″). Thus, the introduction of less than 5 wt% silane acrylate sufficiently controlled the viscoelasticity of the acrylic PSAs prepared with DEGSA or TEGSA. Additionally, the silane acrylates played a role in lowering the modulus of the acrylic PSAs in the low-temperature region (−40 °C), which confirmed that the modulus in the low-temperature region depends on the T_g_ caused by the free volume.

### 3.5. Creep and Stress Relaxation Tests

**Figure 5:**
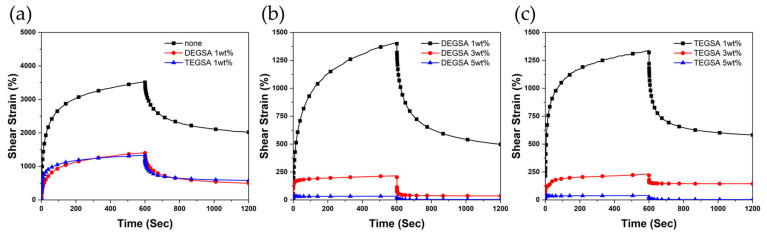
Creep test of acrylic PSA—(**a**) total, silane acrylate 1 wt%, (**b**) DEGSA, (**c**) TEGSA.

Figure 5 shows the creep test results for the acrylic PSAs prepared with the silane acrylates. All acrylic PSAs exhibited a gradual increase in shear strain upon stress application, which is a phenomenon caused by the viscoelastic properties of PSA [22]. As shown in Figure 5, the shear strain of the acrylic PSA without silane acrylate was 3530%, exhibiting a considerable change in strain due to stress. This is because the acrylic PSAs have low cohesion owing to the absence of crosslinking and secondary bonding. The results shown in Figure 4 confirmed that the acrylic PSA without silane acrylate is more viscous than elastic. In general, because PSA is viscoelastic, when an external stress is applied, the PSA behaves in a direction that minimizes the stress, resulting in strain of the PSA. When the viscosity of the PSA is dominant, the strain caused by the external stress is large because the flowability and mobility of the PSA chain are dominant. The addition of 1 wt% silane acrylate reduced the strain of the acrylic PSAs because the elasticity of the acrylic PSAs was improved by silane acrylate. These results are consistent with the modulus characteristics of the acrylic PSAs presented in Figure 4 and confirm that silane acrylate improves the elasticity of acrylic PSAs. When the silane acrylate content was more than 3 wt%, the strain of the acrylic PSAs decreased rapidly, which is owing to the rapid increase in the elasticity of the acrylic PSAs. In conclusion, silane acrylate improved the elasticity of the acrylic PSAs owing to the chain entanglement and bulky silane groups. In addition, it was confirmed that the difference in physical crosslinking due to the silane acrylate chain length also significantly affects the elasticity and creep characteristics of the acrylic PSAs.

**Figure 6:**
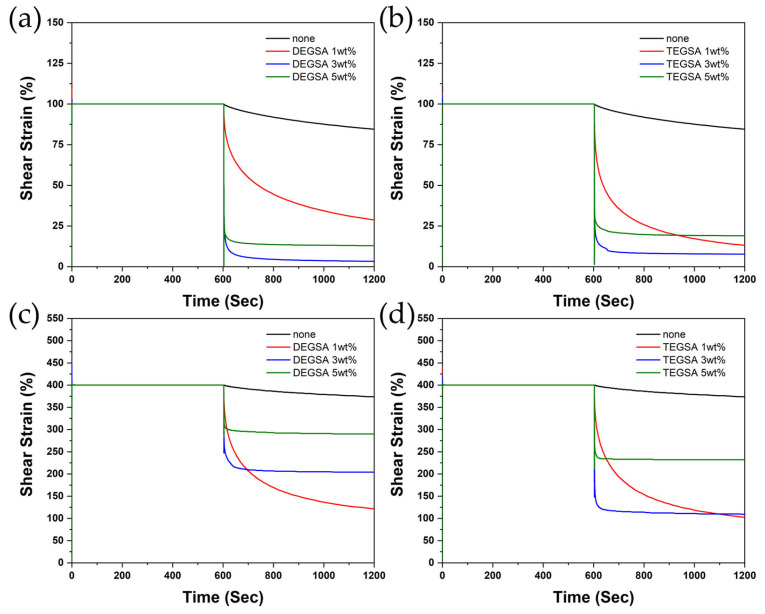
Recovery properties of acrylic PSA—(**a**) DEGSA, strain 100%, (**b**) TEGSA, strain 100%, (**c**) DEGSA, stain 400%, (**d**) TEGSA, strain 400%.

Figure 6 shows the recovery characteristics of the acrylic PSAs prepared with the silane acrylates. When a strain of 100% was applied to the acrylic PSAs, the acrylic PSA without silane acrylate exhibited a very low recovery of 11%, which is owing to the low cohesive force of acrylic PSA [17]. As observed in Figure 1, in the acrylic PSA without silane acrylate, the polymer chain is simply entangled and, thus, the acrylic PSA had low cohesion. When strain is applied to this chain structure, chain entanglement is easily released owing to the insufficient cohesive force between the chains and, thus, the acrylic PSA has low recovery characteristics. As the strain increased from 100% to 400%, the strain recovery characteristics of the acrylic PSA further decreased to 5%; this is because the entanglement of the polymer chain is released at a higher rate by the increased strain. The introduction of silane acrylate significantly improved the recovery properties of the acrylic PSA, and similarly, the recovery properties varied depending on the ethylene glycol chain length. This is owing to the improved elasticity of the acrylic PSA caused by silane acrylate, which significantly increased the recovery property of the acrylic PSA to more than 62%. When the strain of the acrylic PSA was increased from 100% to 400%, the recovery characteristics of the acrylic PSAs prepared with DEGSA and TEGSA decreased gradually. In addition, when the DEGSA and TEGSA content was 5 wt% or higher, the recovery property rapidly decreased to 27%. A rapid increase in elasticity causes a rapid decrease in creep characteristics, and the stress of the acrylic PSA induced by strain increases. Thus, the physical crosslinking due to the entanglement and secondary bonding of the chains in the acrylic PSA is easily broken, thereby reducing the recovery characteristics. Therefore, silane acrylates improved the recovery characteristics by controlling the elasticity of the acrylic PSA, but the rapid increase in elasticity reduced the recovery characteristics.

**Figure 7:**
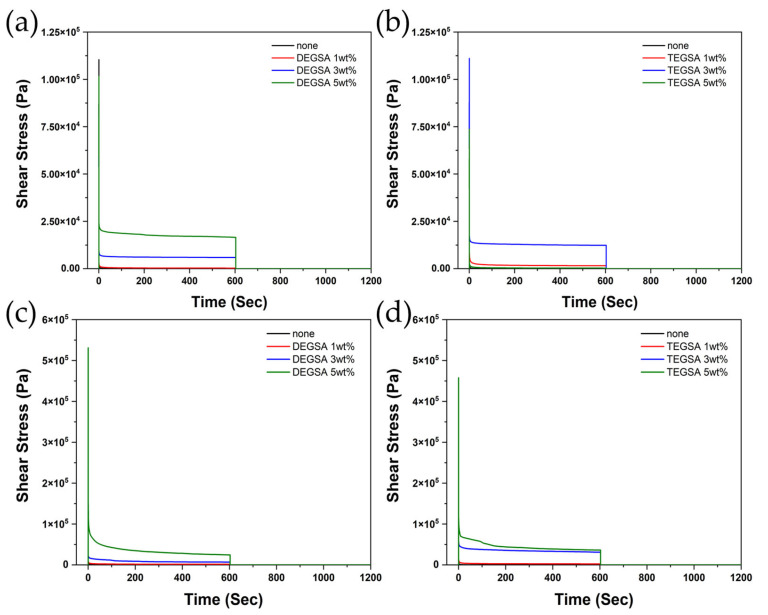
Stress relaxation of acrylic PSA—(**a**) DEGSA, strain 100%, (**b**) TEGSA, strain 100%, (**c**) DEGSA, stain 400%, (**d**) TEGSA, strain 400%.

Figure 7 shows the stress relaxation characteristics of the acrylic PSAs prepared with the silane acrylates. The stress applied to the PSA under 100% strain was 82.6–99.7%, indicating that the PSA has very high relaxation characteristics. This is owing to the very low T_g_ of the synthesized acrylic PSAs. As mentioned previously, the introduction of silane acrylate into the acrylic PSA reduced the T_g_ caused by the free volume and high flexibility was obtained independent of the silane acrylate type. Thus, the acrylic PSA easily induces strain, and the applied stress is relaxed at a high rate due to phenomena such as chain mobility, secondary bond degradation, and loosening of chain entanglement. Moreover, when the strain increased from 100% to 400%, the stress relaxation characteristics increased slightly. Because physical crosslinking is more easily degraded at higher strains, deformation occurs more easily and, thus, the applied stress is more easily relaxed. Consequently, the acrylic PSAs maintained a very low modulus, even upon silane acrylate addition, which contributes to the relaxation of the stress applied to the acrylic PSAs at a very high rate.

### 3.6. Optical Properties

To be applied to flexible optical devices, the optical properties of PSAs should be considered in addition to their adhesion and viscoelasticity [23]. The optical properties of the acrylic PSAs were evaluated using UV–Vis and a haze meter, and the results are shown in Table 3. In general, high transmittance and low haze are required for application to optical devices [24,25]. The synthesized acrylic PSAs prepared with silane acrylates showed excellent transmittance (more than 90%) and very low haze (less than 1%). These properties are attributed to the amorphous structure of acrylic PSA [12], which does not interfere with light movement or cause haze reduction due to scattering because no crystalline region exists. In addition, the sulfur atom in silane acrylate is also thought to enhance the optical properties of acrylic PSAs. Consequently, the synthesized acrylic PSAs prepared with silane acrylates are suitable for application in optical devices owing to their excellent optical properties (transmittance ≥ 90%, haze ≤ 1%).

## 4. Conclusions

In this study, novel silane acrylates (DEGSA and TEGSA) containing ethylene glycol chains were synthesized, and the effect of silane acrylate on the adhesive properties and viscoelastic behavior of the corresponding acrylic PSA was investigated. DEGSA and TEGSA were easily synthesized via a thiol-ene reaction, and the corresponding acrylic PSA was prepared by mixing the silane acrylate with commercial acrylates (EHA and HEA). The introduction of the silane acrylates increased the free volume of the acrylic PSAs owing to the long chain lengths of ethylene glycol. These characteristics increased the molecular weight of the acrylic PSAs and affected their T_g_ values. In addition, the chain entanglement of the acrylic PSAs increased, and physical crosslinking with secondary bonds was induced by ethylene glycol. These properties, in addition to the bulky silane groups, increased the cohesive properties of the acrylic PSAs, resulting in improved adhesive properties. The acrylic PSAs prepared with the silane acrylates exhibited a low modulus of less than 10^6^ Pa, and the modulus in the high-temperature region increased as the silane acrylate content increased. The improvement in elasticity induced by silane acrylate addition was also confirmed by the creep test, and the synthesized acrylic PSAs exhibited similar elasticity independent of the silane acrylate type, above 3 wt% silane acrylate content. The acrylic PSAs prepared with the silane acrylates showed very good recovery characteristics of 62–96% owing to the improved elasticity, and the recovery characteristics decreased as the strain increased. In addition, the synthesized acrylic PSAs exhibited very soft characteristics owing to their low modulus and T_g_ values, and consequently, the acrylic PSAs have excellent stress relaxation characteristics of approximately 90% or higher. In addition, the acrylic PSAs showed excellent optical properties with a transmittance of more than 90% and a haze of less than 1%. Based on these results, the acrylic PSAs prepared with the novel silane acrylates have excellent properties for application in flexible optical devices, independent of the silane acrylate type.

## Figures and Tables

**Table 1:** Mol ratio and synthesis condition of synthesized polymers.

Sample	Acrylate Ratio			PI (wt%)	CTA (wt%)
	EHA	HEA	Silane Acrylate		
EHA	90	10	-	0.5	0.025
DEGSA-1	89		1		
DEGSA-3	87		3		
DEGSA-5	85		5		
TEGSA-1	89		1		
TEGSA-3	87		3		
TEGSA-5	85		5		

**Table 2:** The molecular weight and glass transition temperature of synthesized PSAs.

Sample	Acrylate Ratio			Characterization			
	EHA	HEA	Silane Acrylate	Mn (g/mol)	Mw (g/mol)	PDI	Tg (°C)
EHA	90	10	-	600,000	870,000	1.43	−54.05
DEGSA-1	89		1	630,000	1,110,000	1.75	−53.05
DEGSA-3	87		3	880,000	1,440,000	1.64	−53.96
DEGSA-5	85		5	870,000	1,220,000	1.40	−54.04
TEGSA-1	89		1	800,000	1,160,000	1.45	−51.35
TEGSA-3	87		3	1,050,000	1,350,000	1.39	−53.63
TEGSA-5	85		5	860,000	1,170,000	1.36	−51.48

**Table 3:** Optical peroperties of synthesized PSAs.

Sample	Acrylate Ratio			Optical Properties	
	EHA	HEA	Silane Acrylate	Transmittance (%)	Haze (%)
EHA	90	10	-	91.67	1.41
DEGSA-1	89		1	92.20	1.00
DEGSA-3	87		3	92.44	0.42
DEGSA-5	85		5	92.30	0.73
TEGSA-1	89		1	91.91	0.79
TEGSA-3	87		3	92.36	0.84
TEGSA-5	85		5	92.60	0.63

## Data Availability

Not applicable.

## Supplementary Materials

The following supporting information can be downloaded at: https://www.mdpi.com/article/10.3390/polym15173601/s1, Figure S1: NMR spectrum of synthesized silane acrylates; Figure S2: Surface hydrophilicity of acrylic PSAs according to silane acrylate.
