# Supplementary material for: The Effect of Silane Acrylate Containing Ethylene Glycol Chains on the Adhesive Performance and Viscoelastic Behavior of Acrylic Pressure-Sensitive Adhesives for Flexible Displays
Source: Polymers (Basel). 2023 Aug 30;15(17):3601. doi: 10.3390/polym15173601 (PMC10489647; doi:10.3390/polym15173601)
Supplement: Supplementary file 1 [file polymers-15-03601-s001.zip › polymers-2560222-supplementary.pdf]

# The Effect of Silane Acrylate Containing Ethylene Glycol Chains on the Adhesive Performance and Viscoelastic Behavior of Acrylic Pressure-sensitive Adhesives for Flexible Displays

**Woong Cheol Seok †, Jong Tae Leem † and Ho Jun Song ***

Green and Sustainable Materials R&D Department, Korea Institute of Industrial Technology, 89 Yangdaegiro-gil, Ipjang-myeon, Seobuk-gu, Cheonan-si 31056, Chungcheongnam-do, Republic of Korea; vmfosel07@naver.com (W.C.S.); netwt700@naver.com (J.T.L.)

* Correspondence: song3026@kitech.re.kr; Tel.: +82-41-589-8467; Fax: +82-41-589-8550

† These authors contributed equally to this work.

**Supporting**

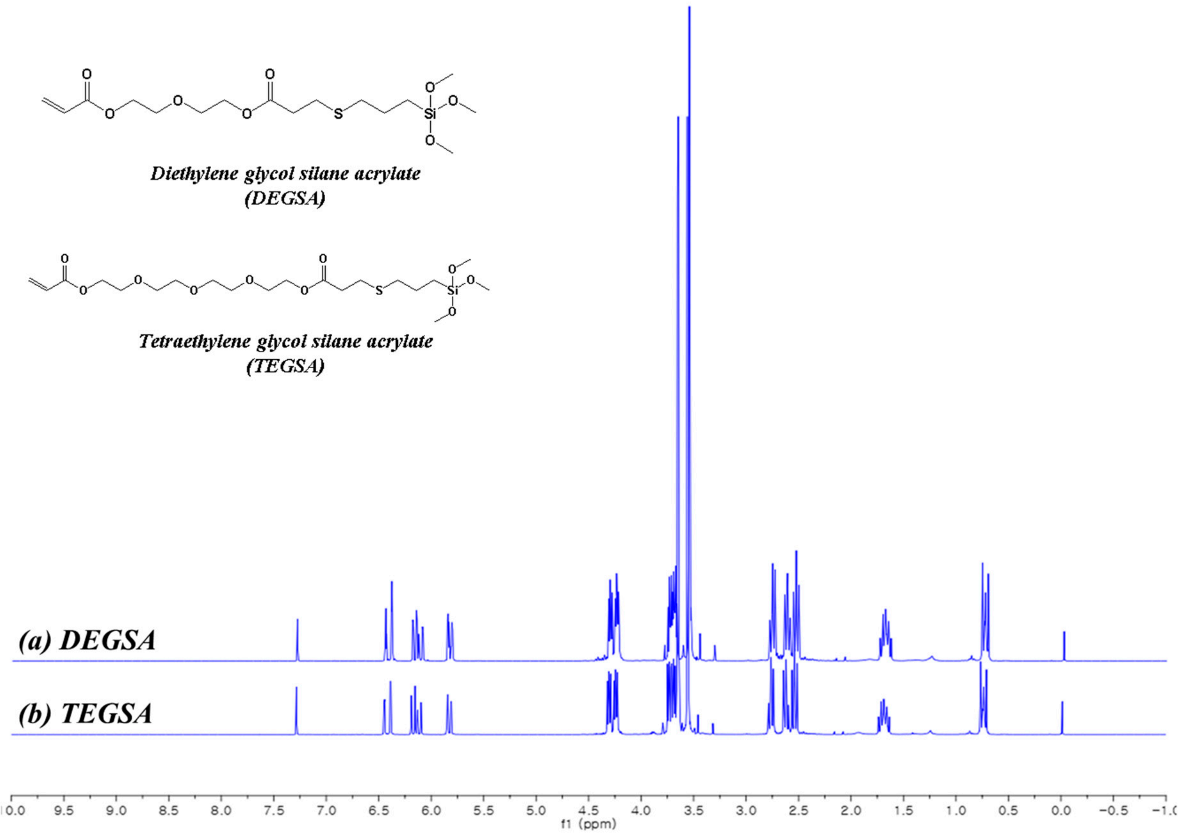


Figure S1 NMR spectrum of synthesized silane acrylates

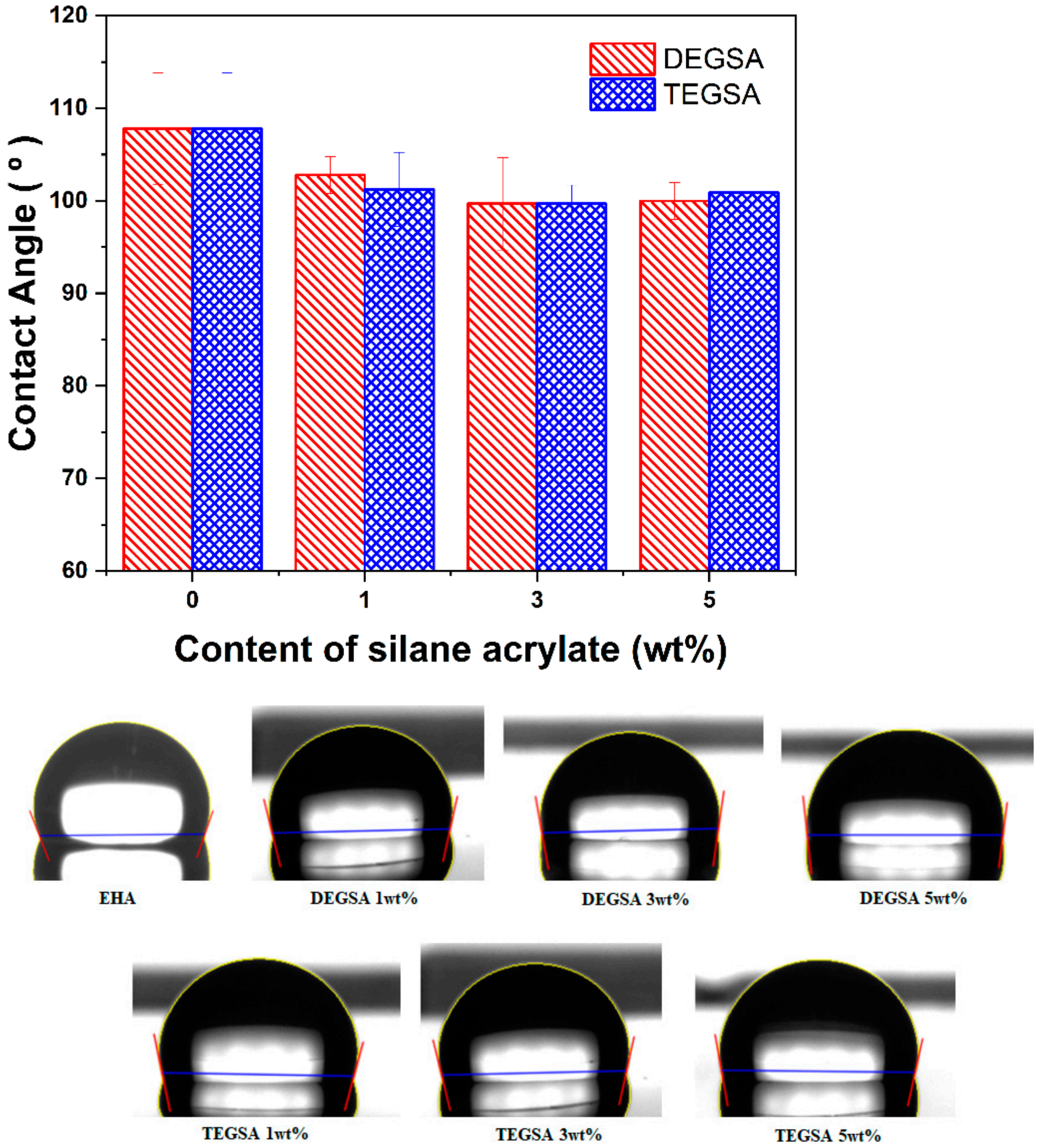


Figure S2 Surface hydrophilicity of acrylic PSAs according to silane acrylate
